# Plastic Surgeon versus Orthopedic Surgeon: Where Would You go for Your Hand Injury? A Cross-Sectional Study in Saudi Arabia

**DOI:** 10.1055/s-0044-1779487

**Published:** 2024-02-07

**Authors:** Muna F. Alnaim, Nouf M. AlRabiah, Hanan Ahmed M Al Kaabi, Basem Zogel, Samar Alfaifi, Nora Ibrahim AlMssallem, Bassmh A. Al Dafer

**Affiliations:** 1 College of Medicine, King Faisal University, Al Ahsa, Saudi Arabia; 2 College of Medicine, King Khalid University, Abha, Saudi Arabia; 3 Faculty of Medicine, Jazan University, Jazan, Saudi Arabia; 4 Division of Surgery, Department of Orthopedic Surgery, College of Medicine, King Faisal University, Al Ahsa, Saudi Arabia

**Keywords:** hand surgery, knowledge and awareness, plastic surgery, Orthopedic surgery, Saudi Arabia

## Abstract

**Background** Hand surgery has become a well-established medical specialty in recent years, with many highly trained hand surgeons practicing in various cities throughout Saudi Arabia. It is crucial to assess the public's knowledge and awareness regarding hand surgery specialists and to identify the existence of bias in the public's perception of plastic and Orthopedic surgeons.

**Methods** A self-administered questionnaire was designed and disseminated to adults in Saudi Arabia via Google Forms. The questionnaire addressed participants' knowledge about which type of surgeon they would consult for various hand-related issues.

**Results** A total of 716 participants were surveyed. Most believed Orthopedic surgeons were more qualified for hand surgeries than plastic surgeons. Furthermore, the public seemed to feel safer with Orthopedic surgeons regarding complications. There was a misconception regarding plastic surgeons' qualifications, with only 24.4% recognizing that all plastic surgeons could perform hand surgery. The majority also held misconceptions regarding Orthopedic surgeons' qualifications for hand surgery. Gender and educational level influenced the responses, with females and those with bachelor's degrees or higher showing slightly more knowledge.

**Conclusion** There is a need for increased public awareness and education regarding the qualifications and capabilities of both plastic and Orthopedic surgeons in hand surgeries. Both specialties are well-trained and competent in this area, and the choice should be based on the specific needs and circumstances of the patient.

## Introduction

One of the most recent specialties to become recognized as a distinct field of study is hand surgery. This specialty was created by general surgeons, plastic surgeons, Orthopedic surgeons, vascular surgeons, and neurosurgeons working together. It was believed to have started with World War II victims. The odd number of survivors with hand injuries has meant that there has been a growing demand for advancement in the treatment of acute injuries that consistently cause late hand deformities. There has been a push for a better understanding of how to handle hand issues. 1 2

As a specialized field, hand surgery depends on general practitioners to diagnose and refer patients. 3 Hand surgery, which is at the intersection of Orthopedic, plastic, and microsurgery, has become a new field of study since treating hand injuries and infections requires expert surgeons trained in certain procedures. 4 Given that hand therapy is crucial for treating a variety of hand problems, a hand therapist spends more time with patients than a surgeon. 5

Hand and upper limb surgery is one of the most difficult subspecialties within Orthopedic and plastic surgery. It takes a profound grasp of the hand's natural structure as well as exceptional skill in correctly detecting and treating the many disorders that can harm the hand in order to guide our patients toward recovering their function. A hand surgeon needs to be unique in a few ways. To be successful, they must possess the best level of technical precision, good surgical skills, an innate artistic ability, knowledge of hand anatomy, be imaginative and creative in order to perform and design new procedures, be delicate enough with the various hand tissues, and pay close attention to every detail. 6

The skill of the hand surgeon is required to manage many hand conditions, such as difficult reconstructive hand surgeries, severe hand infections, mutilating hand injuries, complex congenital hand abnormalities, wrist pathology, microsurgery, malignant tumors, joint replacements, and brachial plexus/peripheral nerve injuries. The area of hand surgery has advanced to the point that many experts have a deep passion for specific subspecialties, such as brachial plexus surgery, microsurgery (including functional muscle transfers), cancer, or wrist surgery. As a matter of fact, prominent centers for these subspecialties of hand surgery are currently in operation. 7 This study aims to assess the general public's knowledge of hand surgery and determine which specialty they associate hand surgery with the most.

## Methods

The authors of this cross-sectional study designed a self-administered questionnaire after analyzing existing literature with similar goals. An Orthopedic surgery expert created and revised the questionnaire to verify the questions' objectivity. The questionnaire was validated by a pilot study consisting of 12 diverse participants and was modified accordingly. In addition to demographic questions, the questionnaire included 13 questions about participants' knowledge of deciding between an Orthopedic and a plastic clinic for a patient with a hand injury.

Participant knowledge calculation was according to the following, out of 13 questions in our study, 8 of them had only one possible answer, depending on the participant's opinion. The participant who answered four out of eight was considered good knowledge, whereas three or fewer were considered unknowledgeable.

All subjects have been informed that no identifiers are needed. Data was protected, and only authorized people had access to it. After institutional review board approval, the survey was distributed using an online questionnaire that is collected through social media for adults in Saudi Arabia. Subjects were asked to complete the survey via Google Forms between October 17 and November 23, 2022. The inclusion criterion was 18 years old and above living in Saudi Arabia who accepted to participate. People outside Saudi Arabia and those who did not prefer to complete the survey were excluded.

### Statistical Analysis

The Statistical Package for Social Sciences (SPSS) version 28 was used for the study's statistical analysis. The data analysis was done using descriptive data analysis, and a *p* -value of 0.05 was considered the cutoff point for the significance level. The sample size was calculated with 95% confidence level and 5% margin of error for the study, revealing a required sample of 385.

## Results

Table 1 shows that 716 participants were involved in this study. Most participants were female ( *n* = 417; 58.2%). More than half of the participants were between 21 and 30 years of age ( *n* = 384; 53.6%), while 13.3, 12.8, and 12.3% patients were aged between 31 and 40, 41 and 50, and 18 and 20 years, respectively. Only 6.6 and 1.4% of the participants were aged between 51 and 70 years. In terms of education, more than two-third (63.8%) patients had bachelor's degrees, followed by 24.9% with high school diplomas, 7.4% with higher education, and only 3.2 and 0.7% in middle and primary school, respectively. About 25.7% of the participants were from the western region, while 23.7% of them were from the northern part, followed by 22.5% of participants from the eastern region, and only 16.8 and 11.3% were from the southern and central areas, respectively. The majority of study respondents (27.9%) were students, followed by 24.2% who were employed, 23.2% who were health studies students, 12.2% who were unemployed, and 6.6 and 6% who were retired or healthcare practitioners.

**Table 1:** The sociodemographic characteristics of our study subjects

Sociodemographic characteristics	*n*	%
Age		
18–20	88	12.3
21–30	384	53.6
31–40	95	13.3
41–50	92	12.8
51–60	47	6.6
61–70	10	1.4
Education level		
Primary school	5	0.7
Middle school	23	3.2
High school	178	24.9
Bachelor degree	457	63.8
Higher education	53	7.4
Gender		
Male	299	41.8
Female	417	58.2
Residential area		
Northern region	170	23.7
Western region	184	25.7
Eastern region	161	22.5
Southern region	120	16.8
Central region	81	11.3
Employment status		
Employed	173	24.2
Unemployed	87	12.2
Student	200	27.9
Health studies student	166	23.2
Healthcare practitioner	43	6
Retired	47	6.6

**Fig. 1:**
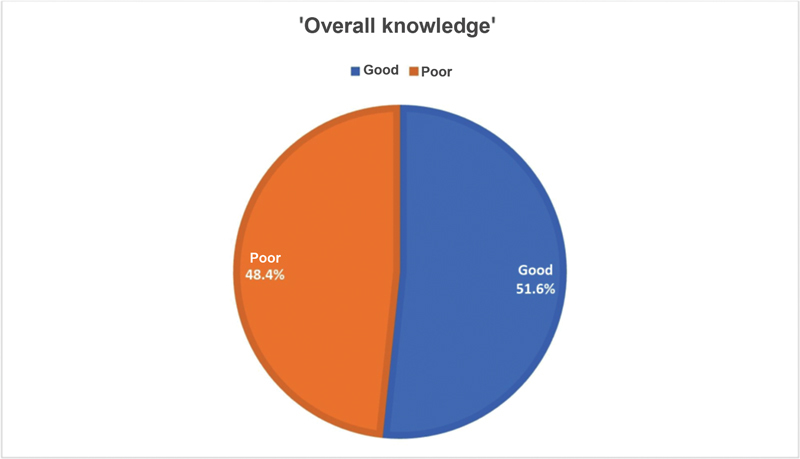
General society knowledge regarding choosing a suitable clinic for a patient with a hand injury.

As shown in Fig. 1 , approximately one-half of the general population had good knowledge regarding choosing a suitable clinic for a patient with a hand injury (51.6%), in comparison to 48.4% who had poor knowledge.

Table 2 provides the results of knowledge regarding choosing a suitable clinic for a patient with a hand injury. When participants were asked about the appropriate clinic for someone born with a congenital hand anomaly, 34.4% knew that a plastic surgeon is a qualified specialist. Approximately half of them said they would go to an Orthopedic surgeon (44.6%), 12.7% said they would visit a general surgeon, and only 8.4% said they did not know. When asked about the doctor who will perform reconstruction when someone's finger gets amputated or deeply cut, most of them knew that they should visit a plastic surgeon (44.6%), followed by an Orthopedic surgeon (39%), and less frequently, a general surgeon (1.9%), while 5.6% said they did not know. About 45.7% believed that Orthopedic surgeons who specialize in hand surgery are qualified to perform reconstruction, while one-quarter did not, and 29.3% did not know. When asked if plastic surgeons are skilled in hand reconstruction surgeries, 44.6% believed that they are, while 28.2% said they did not know. The last reported answer to that question was “I don't know” (27.2%). Most participants felt safer having hand surgery performed by an Orthopedic surgeon (54.9%), 32% felt indifferent, and only 13.1% did not feel that way. Most of them stated that they felt unconcerned about having hand surgery performed by a plastic surgeon (38.1%), approximately one-third felt safer (33.5%), and 28.4% did not think that. When asked multiple-choice questions about specialties that can perform hand surgery, 74.4 and 59.2% had good knowledge, respectively, and they answered Orthopedic and plastic surgery. Neurosurgery was mentioned by 46.1% of respondents, followed by general surgery (32%) and trauma surgery (32%). Only 31.6% knew that not all Orthopedic surgeons could perform hand surgery. About 46.1% believed that all of them were qualified for that, and 22.3% said they did not know.

**Table 2:** Knowledge regarding choosing suitable clinic for a patient with hand injury

Variables	*n*	%
1. When someone is born with a congenital hand anomaly, they will go to		
Orthopedic surgeon	319	44.6
Plastic surgeon	246	34.4
General surgeon	91	12.7
I don't know	60	8.4
2. When someone finger gets amputated or deeply cut, the following doctor will perform reconstruction		
Plastic surgeon	319	44.6
Orthopedic surgeon	279	39
General surgeon	78	10.9
I don't know	40	5.6
3. Orthopedic surgeons who specialize in hand surgery aren't qualified for performing reconstruction		
True	179	25
False	327	45.7
I don't know	210	29.3
4. Plastic surgeons aren't qualified for performing hand reconstruction surgeries		
True	202	28.2
False	319	44.6
I don't know	195	27.2
5. I feel safer having my hand surgery performed by an Orthopedic surgeon		
True	393	54.9
False	94	13.1
Indifferent	229	32
6. I feel safer having my hand surgery performed by a plastic surgeon		
True	240	33.5
False	203	28.4
Indifferent	273	38.1
7. Hand surgery can be performed by which of the following specialities (multiple choices)		
Plastic surgery	424	59.2
Orthopedic surgery	533	74.4
Neurosurgery	330	46.1
General surgery	229	32
Trauma surgery	229	32
8. All Orthopedic surgeons can perform hand surgery		
True	330	46.1
False	226	31.6
I don't know	160	22.3
9. All plastic surgeons can perform hand surgery		
True	175	24.4
False	343	47.9
I don't know	198	27.7
10. When someone get a severe burn in their hand, they go for follow-up to		
Plastic surgeon	543	75.8
Orthopedic surgeon	36	5
General surgeon	109	15.2
I don't know	28	3.9
11. When someone needs joint replacement in the wrist, they go to		
Plastic surgeon	98	13.7
Orthopedic surgeon	553	77.2
General surgeon	40	5.6
I don't know	25	3.5
12. Is the complication rate lowered when an Orthopedic surgeon performs a hand surgery rather than a plastic surgeon?		
Yes	270	37.7
No	140	19.6
I don't know	306	42.7
13. Does a patient have the right to choose what specialty to go for hand surgery? (Orthopedics or plastics surgeon)		
Yes	322	45
No	157	21.9
I don't know	237	33.1

About 24.4% knew that all plastic surgeons could perform hand surgery. While most thought the opposite and 27.7% did not know, most participants were aware that they would go for a follow-up with a plastic surgeon if they got a severe burn on their hand (75.8%). While 15.2% of them mentioned that they would visit a general surgeon, only 5% said they would go to an Orthopedic surgeon, and 3.9% said they did not know. When asked about the best surgeon for the joint replacement in the wrist, the majority (77.2%) knew that they had to go to an Orthopedic surgeon, followed by a plastic surgeon (13.7%), a general surgeon (5.6%), and 3.5% said they did not know. More than one-third of the participants (37.7%) believed the complications rate was lower when the hand surgery was performed by an Orthopedic surgeon rather than a plastic surgeon, 19.6% knew it was not, and the majority (42.7%) had no idea.

Only 21.9% of participants did not know, and 33.1% had no idea that the patient had the right to choose whether to have Orthopedic, plastic, or both types of surgery.

As seen in Table 3 , we compared participants' knowledge with sociodemographic data. A chi-squared test of independence showed no significant association between gender and educational status and knowledge ( *p* = 0.597 and *p* = 0.597, *p* = 0.115). About 59.2% of participants with sound knowledge were female, while only 40.8% were male. More than one-half of the participants with poor knowledge were female (57.3%), and 42.7% were male. Regarding the association between knowledge of hand surgery and education level, the majority of participants who had good knowledge held a bachelor's degree or higher (74%), and only 26% had high school degrees or below. Of those with poor knowledge, 68.6% held a bachelor's degree and above, followed by 31.4% of participants having high school degrees and below.

**Table 3:** The association between hand surgery knowledge and sociodemographic characteristics

Factor	Participant with good knowledge	Participant poor knowledge	*p* -Value
Gender			0.597
Male	141 (40.8%)	158 (42.7%)	
Female	205 (59.2%)	212 (57.3%)	
Education level			0.115
High school degree and below	90 (26%)	116 (31.4%)	
Bachelor degree and above	256 (74%)	254 (68.6%)	

## Discussion

Hand surgery involves the treatment of the hand from the tip to the shoulder. 8 Both plastic and Orthopedic surgeons receive adequate training in hand surgery. 9 Their exposure to the specialty begins during residency and is followed by a 1- to 2-year fellowship in hand surgery, thus making them equally qualified to conduct hand surgery. 10 11 In Saudi Arabia, microsurgery, wrist surgery, and brachial surgery are included in the hand surgery training for fellows. Our study aim is to assess the public knowledge concerning hand surgery clinics. Our study found that the public believed an Orthopedic surgeon was more qualified to perform hand surgery than a plastic surgeon (54.9%). Also, they felt safer in an Orthopedic surgeon's hands regarding complications and believed plastic surgeons could not perform hand surgery. About 24.4% had the assumption that all plastic surgeons could perform hand surgery.

At the same time, the rest believed that they could not, which is unexpected because just under half of the respondents had adequate knowledge of the capabilities of a plastic surgeon. This staggering finding indicates people's lack of knowledge regarding the qualifications of a plastic surgeon. In terms of congenital hand anomalies, a plastic surgeon is an appropriate physician to treat such conditions. However, 44.6% preferred an Orthopedic to treat it. This indicates that the public does not have sufficient information regarding such anomalies. When it comes to reconstruction, the public deemed a plastic surgeon the suitable physician for it. Orthopedics need to be specialized to perform hand surgery, unlike plastic surgeons, who do not need specialization due to their exposure during their residency. Only 31.6% knew that not all Orthopedic surgeons were qualified to perform hand surgery, whereas the majority (46.1%) believed they were.

Regarding wrist joint replacement, the majority (77.2%) agreed that Orthopedic surgeons were the only ones capable of performing such a procedure. In comparison, the rest (13.7%) believed that plastic surgeons could be followed by general surgeons (5.6%). Over one-third (37.7%) thought that the complication rate is lowered when Orthopedic surgeons perform the surgery. The majority did not have enough information to decide, while (19.6%) knew that there was no difference. No evidence in the literature says there is a difference between plastic and Orthopedic surgeons in terms of complication rates. In terms of overall knowledge, females had a slight advantage over males, where they had answered more questions correctly. They seemed to understand both plastic and Orthopedic surgeons' capabilities and roles. Respondents with a bachelor's degree and higher also had more knowledge and awareness about the topic than those with high school diplomas or lower.

Multiple factors contribute to the need for hand surgeons in Saudi Arabia. Congenital anomalies due to consanguineous marriage, tuberculosis manifestations of the hand, and Orf infections are common presentations seen in clinical practice that require a hand surgeon to address and treat. 7 This article tries to clarify the public's misconceptions regarding the qualifications of a plastic surgeon and the favoritism people have toward an Orthopedic surgeon regarding hand surgery. Future research should focus on exploring people's biases concerning different specialties.

### Study Limitations

One of the study's limitations was that the authors did not ask about the source of information the patients received. Also, a higher response rate would be favorable to represent the Saudi population accurately.

## Conclusion

In conclusion, the study found considerable gaps in public understanding regarding the competencies and capabilities of plastic and Orthopedic surgeons in hand surgeries in Saudi Arabia. Although both specialties undergo rigorous training and are equipped to perform hand surgeries, most respondents preferred Orthopedic surgeons. The presence of bias can be attributed to the existence of misconceptions regarding the set of abilities possessed by plastic surgeons. It is evident that many individuals are not fully aware of the wide range of skills that plastic surgeons possess in effectively managing congenital hand anomalies and performing reconstructive procedures. The results also showed that there are demographic disparities in perception, with women and those with bachelor's degrees or higher demonstrating a deeper comprehension. The presence of these misconceptions emphasizes the significance of a thorough public education system while also prompting inquiries into the potential impact of these perceptions on patient decision-making and healthcare results. Further investigation is warranted to explore the underlying factors contributing to these biases, thereby enabling all people to make well-informed choices concerning their healthcare requirements.
